# Managing direct oral anticoagulants in accordance with the Scottish Dental Clinical Effectiveness Programme guidance for patients undergoing dentoalveolar surgery

**DOI:** 10.1038/s41415-022-3999-y

**Published:** 2022-04-22

**Authors:** Sarah A. Woolcombe, Rebecca E. Ball, Jignesh P. Patel

**Affiliations:** 41415105413001 Department of Oral Surgery, King´s College Hospital NHS Foundation Trust, UK; 41415105413002 Department of Haematological Medicine, King´s College Hospital NHS Foundation Trust, UK; Institute of Pharmaceutical Science, King´s College London, UK

## Abstract

**Introduction** The Scottish Dental Clinical Effectiveness Programme (SDCEP) guidance on the management of dental patients taking anticoagulant or antiplatelet drugs provides recommendations on the management of patients taking direct oral anticoagulants (DOACs). This guidance was developed by a multidisciplinary Guidance Development Group, based on available resources at the time of publication. We aim to describe our experience of managing a cohort of adult patients prescribed DOACs, undergoing dentoalveolar procedures in accordance with the SDCEP guidance, between April 2017 and March 2020.

**Methods** As part of our routine practice, patients received a telephone consultation one week following treatment, to assess any post-operative bleeding. Review of the clinical notes was used to assess clinician adherence to the guidance recommendations.

**Results** In total, 98 patients underwent 119 dentoalveolar procedures. Persistent bleeding followed 17 (14.3%) procedures, of which 11 (9.2%) procedures required specific intervention. Absolute compliance with the recommendations was 43.7%, supporting the recommendation for audit and staff education.

**Discussion ** A diagnosis of heart failure and advanced age were identified as contributory factors to post-operative bleeding.

**Conclusions ** The SDCEP guidance is safe to follow, with no patients experiencing major haemorrhage. Consulting a medical specialist for patients with heart failure of likely significance, based on the medical or drug history, in addition to those who report an advanced heart failure diagnosis and the frail/older person, could reduce the incidence of post-operative bleeding.

## Key points

- Details the SDCEP guidance recommendations, at the time of writing, regarding the management of dental patients taking DOACs.
- Informs of the bleeding outcomes for patients taking DOACs who undergo dentoalveolar surgery.
- Assesses the safety and appropriateness of the SDCEP guidance recommendations.
- Highlights the determinants of post-operative bleeding risk.

## Introduction

In our oral surgery department, we treat many patients who are anticoagulated and require dentoalveolar surgery. Post-operative bleeding is at the forefront of any surgeon's mind when performing these procedures due to the risk of associated morbidity and mortality.^1^ At King's College Hospital, direct oral anticoagulant (DOAC) therapy started being prescribed in the summer of 2012. At that time, we had no clinical experience of managing patients undergoing dentoalveolar surgery while prescribed a DOAC and there was no national guidance available. We therefore managed each patient prospectively, on a case-by-case basis, together with the department of haematological medicine.^2^ Haematological investigation was performed for each patient to assess anticoagulation intensity and renal function. This collaborative approach to patient management was formalised as the King's College Hospital (KCH) DOAC pathway. We have previously shared our early experience of this.^2^

The Scottish Dental Clinical Effectiveness Programme (SDCEP) guidance on the management of dental patients taking anticoagulant or antiplatelet drugs was first published in August 2015. To develop the recommendations for this guidance, SDCEP convened a multidisciplinary Guidance Development Group, including medical and dental practitioners, specialists and a patient representative.^3^ The key recommendations presented in the guidance were developed through considered judgements made by the group based on the existing guidelines, the available evidence, clinical experience, expert opinion and patient and practitioner perspectives.^3^ The guidance provides recommendations regarding assessing and managing this group of patients, with a specific section dedicated to the management of patients taking DOACs. Under its key recommendations, the SDCEP guidance suggests that patients undergoing an invasive dental procedure, which is considered to be low risk for bleeding, should be treated with no alteration to their DOAC regime. These procedures include the simple extraction of 1-3 teeth with restricted wound size or an intraoral incision and drainage. The procedures considered to be associated with a higher risk of bleeding include multiple, adjacent extractions with a large wound size, biopsies, flap raising procedures and gingival recontouring.^3^ Patients undergoing these procedures should miss or delay their morning DOAC dose (depending on the specific DOAC prescribed) on the day of their treatment.^3^

The guidance clearly states that these are conditional recommendations with low quality supporting evidence, due to the lack of published data surrounding these drugs and invasive dental procedures. Other studies, including meta-analyses, have also concluded that evidence regarding peri-procedural DOAC management in this setting is lacking and that the quality of studies is poor.^4^^,^^5^^,^^6^ The Guidance Development Group recommends that further research into patients taking DOACs and undergoing dental procedures should be conducted and that compliance with the guidance recommendations should be audited.^3^

The SDCEP guidance also specifies a number of medical conditions and other medications that may increase bleeding risk and advises consulting with a general medical practitioner (GP) or specialist in these cases (Table 1).^3^

**Table 1:** Medical conditions and other medications associated with increased bleeding risk, adapted from the 2015 Scottish Dental Clinical Effectiveness Programme guidance^3^

Medical conditions	Other medications
Chronic renal failure Liver disease Haematological malignancy or myelodysplastic disorder Recent or current chemotherapy Advanced heart failure Inherited bleeding disorders Immune thrombocytopenia (ITP)	Other anticoagulants or antiplatelet drugs Cytotoxic drugs - leflunomide, hydroxychloroquine, adalimumab, infliximab, etanercept, sulfasalazine, penicillamine, gold, methotrexate, azathioprine, mycophenolate Non-steroidal anti-inflammatory drugs (NSAIDs) Drugs affecting the nervous system - selective serotonin reuptake inhibitors (SSRIs), carbamazepine
Note: chronic renal failure is synonymous with chronic kidney disease. The other medications are unrelated to the medical conditions.	

For all patients, it is recommended that treatment should be performed in the morning to allow time for the management of any immediate bleeding complications that occur. In addition, the initial treatment area should be limited, bleeding assessed before continuing and local measures for haemostasis should be applied.^3^ The efficacy of local measures to aid haemostasis is well-supported by the literature.^4^^,^^7^^,^^8^^,^^9^

Since the publication of the SDCEP guidance, we have followed its specific recommendations for those patients identified as being at standard risk of post-operative bleeding. We have continued to manage those patients at increased risk of bleeding, due to their medical or drug history (for whom the guidance recommends seeking a medical opinion) on a case-by-case basis, with input from the anticoagulation team at King's College Hospital.

We assessed the bleeding outcomes for patients who were known to be taking a DOAC and had undergone dental extractions in our department, in accordance with the specific SDCEP directive. Our primary aim was to evaluate the safety of the SDCEP guidance and determine if any modification to the recommendations would be advisable. In addition, we ascertained whether the specific guidance was being consistently applied to the correct cohort of patients and assessed clinicians' compliance with the recommendations through audit.

## Methods

We studied a cohort of 98 consecutive patients who underwent 119 dentoalveolar procedures while prescribed a DOAC, during the period 3 April 2017 to 10 February 2020. All patients had been identified by the assessing clinician as suitable for the specific SDCEP directive, due to their standard risk of bleeding. As part of our routine practice, each patient received a telephone review one week following treatment and this was used, together with information from the clinical notes, to determine the bleeding outcome of the procedure. If a patient did experience bleeding, this was recorded and categorised according to the following:

1. Bleeding present but no action required
2. Required consultation in the oral surgery department but no specific intervention necessary
3. Required surgical intervention (re-suturing and haemostatic packing) and/or the use of a topical antifibrinolytic
4. Required blood transfusion, replacement therapy or desmopressin.

The clinical notes were assessed retrospectively for details of the medical and drug histories. This information was utilised to determine whether each patient had been correctly identified as being at standard risk of bleeding and therefore suitable for management under the specific SDCEP guidance. We also established whether clinicians correctly assessed each procedure pre-operatively as being of low or higher bleeding risk. Clinicians' compliance with other recommendations within the guidance was also reviewed. These included the provision of correct advice to patients regarding their peri-procedural DOAC management, completion of treatment in the morning and the application of full local measures for haemostasis. This service evaluation is registered at The Dental Institute, King's College Hospital NHS Foundation Trust.

## Results

### Demographics of patients and procedures

During the timeframe of this study, 98 patients underwent 119 dental extraction procedures following the specific SDCEP guidance. The age of these patients ranged from 42-91 years, with a mean age of 70 years. Of these, 48.0% (n = 47) were women. The patients were taking a range of types and doses of DOAC drugs, the most frequently prescribed being rivaroxaban 20 mg once daily (OD) (n = 45) and apixaban 5 mg twice daily (BD) (n = 27) (Table 2).

**Table 2:** Information on concurrent DOAC types and doses

**DOAC prescribed (N = 98)**	**N (%)**
Rivaroxaban 20 mg OD	45 (45.9)
Rivaroxaban 15 mg OD	3 (3.1)
Rivaroxaban 10 mg OD	3 (3.1)
Rivaroxaban unknown dose OD	1 (1.0)
Apixaban 5 mg BD	27 (27.6)
Apixaban 2.5 mg BD	9 (9.2)
Edoxaban 60 mg OD	7 (7.1)
Edoxaban 30 mg OD	2 (2.0)
Dabigatran 110 mg BD	1 (1.0)

Of the procedures performed, 103 were routine dental extractions and 16 were surgical extractions. In total, 65 cases involved extraction of single teeth, and in 54 cases, multiple teeth were extracted - in most cases, two or three (Table 3).

**Table 3:** Types of anaesthesia and procedures performed

**Procedure and anaesthesia type (N = 119)**	**N (%)**
**Anaesthesia type**	
Infiltration	79 (66.4)
Block	40 (33.6)
**Procedure**	
Routine	103 (86.6)
Surgical	16 (13.5)
Single	65 (54.6)
Multiple	54 (45.4)

### SDCEP specific guidance eligibility

Of the 98 patients taking DOACs who were treated according to the specific SDCEP directive, 36 patients (45 procedures) were identified in retrospect as, in fact, having been unsuitable for application of these recommendations. The guidance recommends seeking a medical opinion in these cases, due to medical conditions and/or concurrent drugs which increase the risk of bleeding, the presence of which became apparent on review of the clinical notes (Table 4).

**Table 4:** Medical conditions and concurrent drugs precluding suitability for the specific SDCEP directive

**Specific SDCEP guidance inappropriate (N = 36)**		**N (%)**
**Medical conditions**	Heart failure (not reported by patient)	23 (23.5)
	Heart failure (reported by patient)	5 (4.2)
	Chronic liver disease	1 (1.0)
	Chronic kidney disease	1 (1.0)
**Concurrent drugs**	NSAIDs	1 (1.0)
	Antiplatelets	3 (3.1)
	SSRIs (eg citalopram, sertraline)	4 (4.1)
	Cytotoxic drugs (eg methotrexate, hydroxychloroquine)	3 (3.1)
Note: four patients had multiple comorbidities making them unsuitable for the specific SDCEP guidance.		

Over the course of the study, there were an additional 190 patients who were correctly assessed as being unsuitable for application of the specific SDCEP directive. These patients were managed in accordance with the King's College Hospital DOAC pathway with advice from the anticoagulation team constituting the recommended specialist opinion. This equates to 21.5% of all patients taking DOACs treated during this timeframe being eligible for the specific SDCEP instruction.

### Bleeding outcomes

**Fig. 1:**
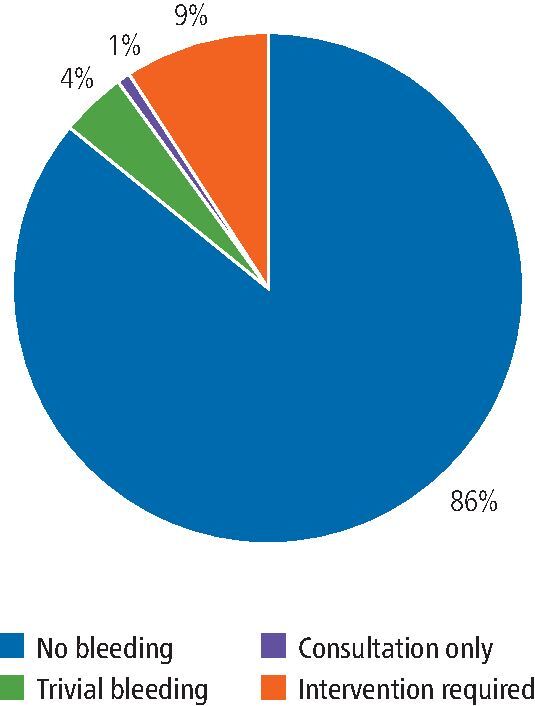
Post-operative bleeding outcomes for procedures performed following the specific SDCEP guidance

In our cohort, where the specific SDCEP directive was followed, there was no post-operative bleeding following 102 (85.7%) procedures. Post-operative bleeding followed 17 (14.3%) procedures, of which five (4.2%) procedures had trivial bleeding requiring no specific intervention. Consultation in the oral surgery unit was required following one (0.8%) procedure, but no intervention was necessary. Specific intervention (re-suturing and haemostatic packing) and/or topical use of an antifibrinolytic agent was indicated to arrest bleeding which occurred following 11 (9.2%) procedures (Fig. 1). No patients required systemic antifibrinolytics, blood transfusion, replacement therapy or desmopressin.

### Contributory factors to bleeding

Of the 11 patients (11 procedures) who experienced significant post-operative bleeding requiring surgical intervention, six (55%) were determined in retrospect to have been unsuitable for application of the specific SDCEP instruction. Four of these patients had significant heart failure (HF) which they had not reported in the medical history. This was identified retrospectively from the drug history or elucidated from patient reports of cardiac amyloidosis/an implanted cardiac resynchronisation therapy defibrillator (CRT-D) device. Two patients had a diagnosis of significant heart failure which they had reported in the medical history. One of these patients had also reported liver disease and chronic kidney disease (CKD) as comorbidities and another was taking concurrent clopidogrel.

**Fig. 2:**
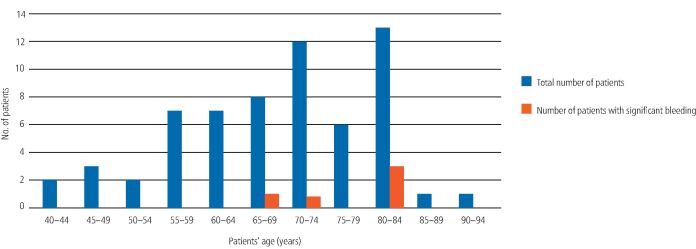
Age distribution of patients who were appropriate for the specific SDCEP guidance

There were five patients (five procedures) who were confirmed as having been appropriate for application of the specific SDCEP directive but who nevertheless experienced bleeding requiring surgical intervention post-operatively. This group accounted for 45% of the patients who experienced significant bleeding. It was noted that these patients were all older adults, three of whom were aged 80 years or older, one of whom was 71 years and one of whom was 67 years (Fig. 2). One of these patients was prescribed oral antimicrobials when they attended post-operatively. However, bleeding restarted within 24 hours of treatment in this patient, so is unlikely to have occurred as a result of infection. Two of these patients had procedures completed in the early afternoon but treatment was otherwise fully compliant with all other aspects of the guidance. Completing procedures in the afternoon had the potential to impact on bleeding management but would not have increased the risk of bleeding occurring. There were no contributory factors to bleeding, apart from advanced age, identified in these cases. The proportion of patients aged 65 years or older, in the cohort of 62 patients correctly assessed as appropriate for the specific SDCEP guidance, was 66.1% (n = 41). The mean age of those who experienced significant bleeding (76 years) was found to be significantly higher than the mean age of the specific SDCEP-guidance-appropriate group as a whole (69 years) using a single sample t-test at the p <0.05 significance level (*t* = 2.4668; p = 0.0346).

### Presentation and management of bleeding

**Fig. 3:**
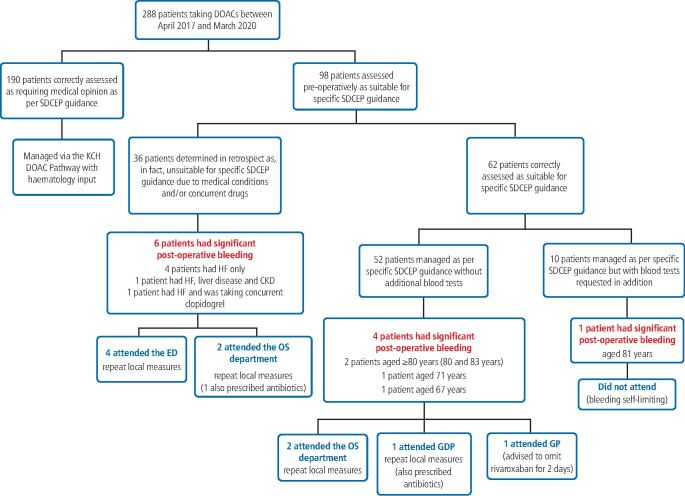
Summary of patient management and bleeding outcomes

Four of the patients (four procedures) who experienced significant post-operative bleeding attended the emergency department (ED) for specific intervention. These patients all had a diagnosis of heart failure and one patient also had comorbid liver disease and CKD. Four patients (four procedures) had specific intervention performed in the oral surgery department and one patient (one procedure) attended their general dental practitioner (GDP). Two patients (two procedures) were additionally prescribed oral antimicrobials as infection was suspected to be a contributory factor to secondary haemorrhage. One patient (one procedure) attended their GP regarding post-operative bleeding. No surgical intervention was performed but they were advised to omit rivaroxaban for two days. One patient (one procedure) experienced persistent bleeding over several days and would have benefited from specific intervention, but they did not attend. In this case, the bleeding was self-limiting without significant morbidity (Fig. 3).

### Timing of bleeding

Bleeding recommenced within 24 hours for seven of these cases. Two patients restarted bleeding after two days, one at six days and another at seven days.

### Adherence to SDCEP guidance

The SDCEP guidance was only followed completely accurately by clinicians in 43.7% of cases (n = 52). In the remaining 56.3% of cases (n = 67), the guidance was not followed precisely. Patients who were incorrectly determined to be at standard bleeding risk and suitable for the specific SDCEP directive included 23 patients (30 procedures) with significant heart failure, which was not reported by the patient, but could have been elucidated from the medical or drug history and five patients (five procedures) who did report a heart failure diagnosis. There was also one patient (one procedure) who reported both liver disease and chronic kidney disease as comorbidities, in addition to heart failure (Table 4).

In total, 11 patients (15 procedures) were incorrectly managed according to SDCEP recommendations when taking concurrent drugs, which the guidance specifies as increasing bleeding risk (Table 4). Our cohort also included one patient who was taking the serotonin and noradrenaline reuptake inhibitor (SNRI) venlafaxine. Although not stipulated in the 2015 SDCEP guidance, SNRIs have a similar ability to potentiate bleeding as selective serotonin reuptake inhibitors (SSRIs).

Overall, 16 patients (16.3%) who underwent 18 (15.1%) procedures were given incorrect advice regarding adjustment to their peri-procedural DOAC regime. In most cases, patients were advised to omit more DOAC doses than the guidance recommends. Moreover, two (2.0%) patients who underwent two (1.7%) procedures had no advice regarding their peri-operative DOAC management, documented in their medical records.

In total, 76 patients (77.6%) who underwent 95 (79.8%) procedures followed the clinician's advice regarding management of their DOAC peri-procedurally. The remaining 22 patients (22.4%) who underwent 24 (20.2%) procedures elected to omit DOAC doses against the clinician's advice.

In 85.7% of cases (n = 102), the clinician correctly assessed the planned procedure as being either low or higher risk of bleeding, according to SDCEP categorisation, at the pre-operative consultation. In 13.4% of cases (n = 16), the procedure performed had been incorrectly assessed pre-operatively as being of higher bleeding risk. In one of the cases (0.8%), the planned procedure had been incorrectly determined to be of low bleeding risk. Retrospective review of the clinical notes and pre-operative radiograph for this case concluded that the likelihood of it becoming a higher bleeding risk procedure (surgical removal of a lower third molar) was predictable from the information available to the assessing clinician. This patient did not experience any post-operative bleeding, however, perhaps because they elected to omit both doses of apixaban on the day of treatment, against the clinician's advice.

The SDCEP guidance recommends that invasive procedures should be carried out early in the day. Our investigation found that 66.4% of procedures (n = 79) were performed in the morning and 33.6% of procedures (n = 40) were carried out in the afternoon. However, 27.5% of the afternoon procedures (n = 11) were completed between 12.00-12.40 pm. Patients who required emergency treatment on the day of presentation account for some procedures that were performed later in the afternoon.

Local measures for haemostasis, comprising haemostatic packing and suturing of the socket were documented as having been applied in full for 95.0% of procedures (n = 113).

**Fig. 4:**
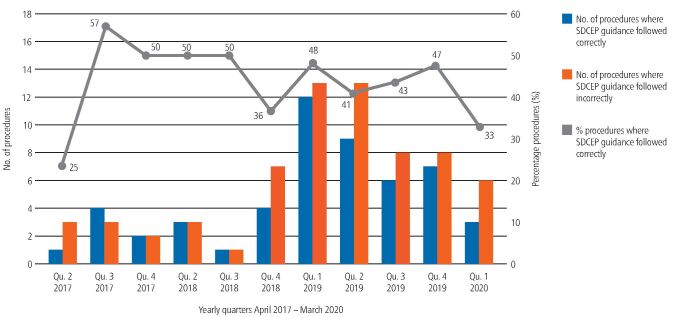
Clinicians' absolute adherence to the SDCEP guidance recommendations over time. (Note: there were no patients taking DOACs treated in quarter 1 [January - March] 2018)

Meticulous adherence to all the recommendations within the SDCEP guidance was not demonstrated to have improved over time, despite increasing clinician familiarity with the guidance. Between 2017-2020, precise compliance with all the recommendations ranged between 25-57% (Fig. 4).

There were ten patients (12 procedures) who were correctly identified as being suitable for the specific SDCEP directive and were managed in accordance with these instructions, but for whom haematological investigation was also requested, despite not being indicated. These patients all had DOAC concentrations considered in range, in line with published data. Specialist haematology opinion, following assessment of anticoagulation intensity and renal function, concurred with the specific SDCEP recommendations for all patients in this group.

## Discussion

The SDCEP guidance was developed to aid clinicians providing invasive dental treatment with the management of patients taking DOACs. It is therefore advantageous for the specific recommendations to be applicable to as many patients in this category as possible. In our study, only 21.5% of all patients taking DOACs, who were treated in the oral surgery department, were appropriate for application of the specific SDCEP directive, without specialist medical consultation being advised. Although this proportion may seem relatively small, as a tertiary referral centre, the majority of our patients have been referred on the basis of medical and/or surgical complexity. It is therefore highly likely that the specific SDCEP recommendations are appropriate for a much larger proportion of patients prescribed DOACs who present for treatment in primary care.

**Fig. 5:**
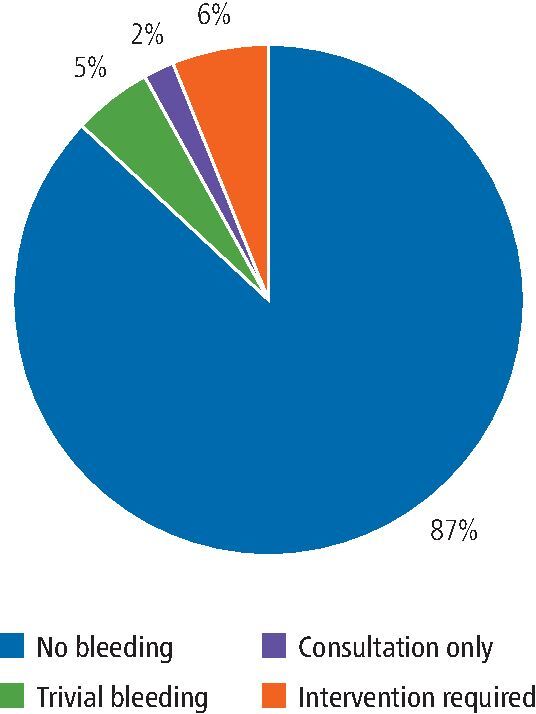
Post-operative bleeding outcomes for procedures performed following individualised, specialist haematology advice, including blood sampling

The incidence of post-operative bleeding complications, for patients managed in line with the specific SDCEP recommendations, followed the same distribution as our previously published outcomes for patients managed with specialist input, prior to our adoption of the SDCEP guidance (Figures 1 and 5).^2^

It was noted that significant bleeding followed 9.2% of procedures managed according to the specific SDCEP instruction, compared to 6.3% of procedures managed under direction of the haematology team, from our previous work.^2^ This may be an expected disparity between the implementation of generalised recommendations and patient-specific, specialist input, which included blood sampling. The difference was not found to be statistically significant (*x*^2^ = 0.6869; p = 0.4072); however, any observed difference in bleeding outcome warrants consideration due to potential morbidity and mortality. As no patients experienced major haemorrhage, this higher incidence of bleeding may be considered an acceptable compromise, if the requirement for specialist advice is negated.

Our study did, however, identify a number of factors that may account for the difference in observed bleeding outcomes. Of the 98 patients managed according to specific SDCEP recommendations, 36 patients were identified in retrospect as, in fact, being unsuitable for the specific instruction, due to medical conditions and/or concurrent drugs which may potentiate bleeding (Table 4). The medical condition most frequently overlooked was significant heart failure and there are a number of possible explanations for this. Overall, 23 of the 28 patients with heart failure did not report this in the medical history. The diagnosis of heart failure was identified from retrospective review of the drug history or elucidated from patient reports of cardiac amyloidosis/an implanted CRT-D device. Determining the presence of heart failure from the drug history is not straightforward, as many drugs used in the treatment of heart failure are also used to treat hypertension or are prescribed post ST-segment elevation myocardial infarction. The use of furosemide and/or an aldosterone antagonist (for example, spironolactone or eplerenone) is, however, highly suggestive of a significant heart failure diagnosis. Similarly, the presence of an implanted CRT-D device or cardiac resynchronisation therapy pacemaker (CRT-P) device is also indicative of significant heart failure. The SDCEP guidance specifies 'advanced heart failure' as a condition associated with increased bleeding risk. Even when patients report a heart failure diagnosis, it is difficult to ascertain its severity unless the patient is cognizant of this. Patients often report their comorbidities as 'well controlled', which could accurately describe advanced heart failure, subject to stringent drug management. Determining the severity of heart failure is complex as it depends upon a multitude of factors. These include patient symptoms and signs (dyspnoea, fatigue, oedema), chest radiograph signs (cardiomegaly and pleural effusions), echocardiogram findings and plasma B-type natriuretic peptide levels.^10^ In addition, the pathophysiology of increased bleeding risk, secondary to underperfusion of the liver as a result of heart failure, may not be intuitive to all clinicians. The presence of heart failure does, however, appear to be significant. All the patients who were incorrectly assessed as suitable for application of the specific SDCEP recommendations and who experienced significant bleeding post-operatively, had a heart failure diagnosis identified in retrospect. In addition, patients with heart failure accounted for all four attendances at the emergency department with post-operative bleeding.

There were also a number of patients taking concurrent drugs associated with increased bleeding risk. Of these, the drugs which were most frequently overlooked were the SSRIs. These drugs potentiate bleeding by inhibiting the uptake of serotonin into platelets, leading to impairment of platelet aggregation.^11^ We also noted that one patient, who experienced minor post-operative bleeding, was taking the SNRI venlafaxine in combination with aspirin. Although not specified in the 2015 SDCEP guidance, SNRIs also inhibit platelet serotonin uptake and studies have shown them to significantly increase bleeding risk.^12^^,^^13^

In total, 45% of patients who experienced significant post-operative bleeding were confirmed retrospectively as appropriate for application of the specific SDCEP recommendations, as they currently stand. It was noted that these patients were all older adults, three of whom were aged 80 years or older, one of whom was 71 years and one of whom was 67 years (Fig. 2). The increased risk of bleeding in this group was found to be independent of comorbidities or polypharmacy and is likely to be associated with age-related renal impairment, particularly in those who are frail.^2^ For this reason, it may be advisable to amend the guidance to suggest consulting a physician for all patients over the age of 80 years and for patients over the age of 65 years, if there is concomitant frailty. Interestingly, our findings are congruent with the HAS-BLED scoring system, which specifies age >65 years as one of the determinants used to estimate long-term risk of major bleeding in anticoagulated patients with atrial fibrillation (Table 5).^14^

**Table 5:** The HAS-BLED scoring system used to assess one-year risk of major bleeding in anticoagulated patients with atrial fibrillation (score 3 or above indicates high risk)

**Letter**	**HAS-BLED risk factor**	**Points**
H	Hypertension (>160 mmHg systolic)	1
A	Abnormal liver or renal function (one point each)	1 or 2
S	Stroke	1
B	Bleeding history or predisposition	1
L	Labile international normalised ratio (INR)	1
E	Elderly (age >65 years)	1
D	Drugs (antiplatelet or NSAID) or alcohol (one point each)	1 or 2
**Maximum score**		**9**

It would certainly seem prudent to minimise the risk of post-operative bleeding in this patient cohort. Older individuals may be more sociably vulnerable, experience relative difficulty in accessing emergency care and have age-related physiological changes, which render them less able to compensate for hypovolemic volume depletion. Any post-operative bleeding risk reduction would, however, have to be carefully balanced against the burden of seeking a medical opinion for all older patients, particularly in the primary care setting.

Our post-operative bleeding rates compare favourably to those reported in the literature, which vary from 3-28.6% and have been observed for up to 13 days post dental extraction. In these studies, some patients continued taking DOACs as per usual and some omitted DOAC doses prior to treatment.^2^^,^^4^^,^^7^^,^^15^^,^^16^^,^^17^^,^^18^

For those patients who were eligible for the specific SDCEP directive but had additional assessment of their anticoagulation intensity and renal function by blood sampling, specialist opinion concurred with the SDCEP recommendations, supporting their safety and appropriateness.

Clinicians demonstrated a high level of accuracy in predicting whether the planned procedure would have a low or higher risk of bleeding. In those cases where the procedural bleeding risk was misjudged, in all but one case the surgeon had erred on the side of caution.

Clinicians' complete adherence to the SDCEP guidance was found to be generally low, ranging from 25-57% over time (mean 43.7%) (Fig. 4). While this may reflect the relative complexity of the recommendations, it does support the Guidance Development Group's recommendation for audit of compliance and the need for regular staff education.

Patients are themselves cautious with regard to their peri-procedural anticoagulation, often electing to omit more DOAC doses than advised. To our knowledge, no pathological thrombotic events occurred as a result of omitting DOAC doses during the course of our study, even when more doses were omitted than the guidance recommends. In the Ockerman trial, however, one patient had a transient ischaemic attack following interruption of DOAC therapy for two days, in preparation for a dental extraction.^18^

Edoxaban first became available for use in the UK in July 2015 and is notably omitted from the 2015 SDCEP guidance, which was published shortly thereafter.

The results of our study should be considered in the context of its limitations. Despite being a large oral surgery unit, our cohort of patients undergoing dentoalveolar surgery, while prescribed a DOAC, is relatively small and our findings are those of a single centre.

## Conclusions

Our findings support the safety and appropriateness of the SDCEP guidance for the management of patients taking DOACs. We consider it highly unlikely that a patient would experience major post-operative haemorrhage when managed in accordance with the guidance, although clinicians should ensure they are familiar with the full recommendations and follow them accurately.

It can be anticipated that approximately 9% of patients who undergo dentoalveolar surgery according to the specific SDCEP directive will re-attend for repeated application of local measures for haemostasis, in order to manage persistent bleeding. This figure may be somewhat reduced, with heightened clinician vigilance with regard to the medical history and some minor modifications to the SDCEP recommendations as they currently stand.

We would advocate consulting a medical specialist for patients with heart failure of likely significance based on the medical or drug history, in addition to those who specifically report an advanced heart failure diagnosis. This should include patients taking furosemide and/or an aldosterone antagonist and those who report an implanted CRT-D/CRT-P device or cardiac amyloidosis.

The guidance could suggest consulting a GP or medical specialist for patients over the age of 80 years and those over the age of 65 years if there is extreme concomitant frailty, providing this would not place undue burden on primary care practitioners.

We recommend a minor addition to the guidance to include SNRIs, such as venlafaxine and duloxetine, in the list of concurrent drugs that place patients at increased risk of bleeding.

Finally, we suggest that the guidance be updated to include edoxaban, together with rivaroxaban, as a once daily DOAC, whose morning dose should be delayed for dental procedures with a higher risk of bleeding complications.
